# Central Antinociceptive and Mechanism of Action of *Pereskia bleo* Kunth Leaves Crude Extract, Fractions, and Isolated Compounds

**DOI:** 10.1155/2015/915927

**Published:** 2015-07-27

**Authors:** Carolina Carvalho Guilhon, Ikarastika Rahayu Abdul Wahab, Fabio Boylan, Patricia Dias Fernandes

**Affiliations:** ^1^Federal University of Rio de Janeiro, Institute of Biomedical Science, Avenida Carlos Chagas Filho 373, CCS Building, 21941-902 Rio de Janeiro, RJ, Brazil; ^2^Faculty of Agro Industry and Natural Resources, Universiti Malaysia Kelantan, 15400 Kota Bharu, Kelantan, Malaysia; ^3^School of Pharmacy and Pharmaceutical Sciences and Trinity Biomedical Sciences Institute, Trinity College Dublin, Dublin 2, Ireland

## Abstract

*Pereskia bleo* (Kunth) DC. (Cactaceae) is a plant commonly used in popular medicine in Malaysia. In this work, we evaluate the antinociceptive effect of* P. bleo* leaf extracts and isolated compounds in central antinociceptive model. Ethanol extract (E), hexane (H), ethyl acetate (EA), or butanol (B) fractions (30, 50, or 100 mg/kg, p.o.), sitosterol (from hexane) and vitexin (from ethyl acetate), were administered to mice. Antinociceptive effect was evaluated in the hot plate and capsaicin- or glutamate-induced licking models. Morphine (1 mg/kg, p.o.) was used as reference drug. Naloxone (1 mg/kg, i.p.), atropine (1 mg/kg, i.p.), and L-nitro arginine methyl ester (L-NAME, 3 mg/kg, i.p.) were administered 30 min earlier (100 mg/kg, p.o.) in order to evaluate the mechanism of the antinociceptive action. Higher dose of B developed an effect significantly superior to morphine-treated group. Naloxone prevented the antinociceptive effect of all fractions. L-NAME demonstrated effect against E, EA, and B. In all fractions, sitosterol and vitexin reduced the licking time after capsaicin injection. Glutamate-induced licking response was blocked by H, EA, and B. Our results indicate that* Pereskia bleo* fractions, sitosterol and vitexin, possessed a central antinociceptive effect. Part of this effect is mediated by opioid receptors and nitrergic pathway.

## 1. Introduction

Pain is a major cause of distress, both physical and psychological, and is also associated with increased inpatient hospital stay, poor wound healing, and prolonged rehabilitation. Opioids and nonopioids are the major classes of analgesic drugs used for the relief of pain. However, while these agents are effective, they are associated with considerable adverse effects, including ulcers, nausea, vomiting, pruritus, tolerance, confusion/hallucinations, respiratory depression, and constipation. Additionally, although effective for acute pain, analgesia is of limited effectiveness for chronic and neuropathic pain states, such as phantom limb pain [1]. These situations lead to the use of alternative approaches to alleviation of the pain states.

*Pereskia bleo* (Kunth) DC. (Cactaceae) is a plant commonly used in popular medicine by the local communities in Malaysia for the treatment of rheumatism, inflammation, gastric pain, ulcers, diabetes, and hypertension and for revitalizing the body and as natural remedy for cancer-related diseases [2–4]. Previous phytochemical studies identified four alkaloids, 3,4-dimethoxy-*β*-phenethylamine, mescaline, 3-methoxytyramine, and tyramine [5]. Kaempferol and quercetin were also isolated [6]. Wahab et al. [7] isolated phytol, *β*-sitosterol, 2,4-di-tert-butylphenol, and vitamin E while Malek et al. isolated campesterol, stigmasterol, dihydroactinidiolide, and 2,4-di-tert-butylphenol in the same year.

Recently our group isolated for the first time vitexin and *β*-sitosterol glucoside from the fraction obtained from leaves of* P. bleo*. We also demonstrated that these fractions have anti-inflammatory effect and antinociceptive activity in inflammatory and peripheral models [8].

In the present work, we decided to go further and evaluate the antinociceptive effect of* P. bleo* leaves' ethanol extract and its fractions as well as sitosterol and vitexin, isolated from hexane and ethyl acetate fractions, respectively, using the central antinociceptive model, hot plate, followed by the investigation of the possible mechanism of action using some antagonists as well as their response on capsaicin- and glutamate-induced licking.

## 2. Materials and Methods

### 2.1. Plant Material and Extract and Fractions Preparation

*Pereskia bleo* leaves (3.5 kg) were collected in Kota Bharu (Kelantan, Malaysia) in August 2009. A voucher specimen is deposited in the Herbarium of the Department of Chemistry, Faculty of Science, University of Malaya, Kuala Lumpur, Malaysia, under the number KL5,729.

The ethanol extract (E) prepared from leaves (1.4 g) was evaporated, suspended in water, and submitted to liquid-liquid partition with solvents of increasing polarity: hexane (H), ethyl acetate (EA), and butanol (B). After several chromatographic procedures, sitosterol was isolated from the hexane fraction and vitexin was isolated from the ethyl acetate fraction [8].

### 2.2. Animals

The animals used in this study were male Swiss mice (20–25 g) obtained from Instituto Vital Brazil. Animals were held in circadian cycles in a temperature-controlled room (22 ± 2°C), with free access to food and water. Twelve hours before the assays, animals were limited to a nonfood diet in order to abolish food interaction with extracts administered, avoiding the decreasing of substance absorption. Animal care and research protocols (ICBDFBC-015) were in accordance with the principles and guidelines adopted by the Brazilian College of Animal Experimentation (COBEA) and were approved by the Ethical Committee for Animal Research (Biomedical Science Institute/UFRJ).

### 2.3. Drugs and Extracts Administration

L-Nitro-arginine methyl ester (L-NAME), capsaicin, and atropine were purchased from Sigma (St. Louis, MO, USA). Morphine hydrochloride was purchased from Merck Inc. (Brazil) and naloxone from Cristália (São Paulo, Brazil). All drugs were dissolved in phosphate buffer saline (PBS) just before use. The ethanol extract and fractions were dissolved in sterile water and administered by oral gavage at doses of 30, 50, and 100 mg/kg in a final volume of 0.1 mL. Morphine (5 mg/kg) was used as the reference drug and was also administered via oral gavage. The negative control group was composed of mice given the vehicle (sterile water); significant effects due to the water* per se* were not observed throughout the study. The dose of morphine was chosen based on previous experiments done by our group [9] and was the one that caused 50% reduction on each protocol (IC_50_).

### 2.4. Hot Plate Test

The hot plate test was, in certain ways, similar to that one described by Sahley and Berntson [10], with some modifications adopted by Matheus et al. [11]. Mice were put on a hot plate (Insight Equipment, Brazil) with the temperature set at 55 ± 1°C. The nociceptive reaction time was measured when the animals started to lick or shake their forepaws or hind paws and/or jumped, at intervals of 30 min following oral administration of vehicle, morphine, or the extracts. Baseline levels were defined as the mean of the reaction times obtained at 60 and 30 min prior to administration of vehicle, extracts, or morphine. Antinociception was quantified as either the increase in baseline (%), calculated by the formula (reaction time × 100/baseline) − 100, or the area under the curve (AUC) defining the interval from 30 to 150 min following drug administration. A formula based on the trapezoid rule was used to calculate the AUC [AUC = 30 × IB[(min⁡30)+(min⁡60)+⋯+(min⁡150)/2], where IB is the increase from baseline (%)].

### 2.5. Capsaicin and Glutamate Test

Aiming to elucidate the antinociceptive response of* P. bleo* fractions, vitexin and sitosterol, the participation of vanilloid receptor (TRPV1) in this study was verified by performing the capsaicin test according to Sakurada et al. [12]. The participation of glutamate receptors was evaluated using the method described by Beirith et al. [13]. The animals were treated with vehicle E, H, EA, and B (30, 50, and 100 mg/kg, p.o.) or sitosterol and vitexin (1, 3, and 10 mg/kg, p.o.) one hour before the intraplantar injection of 20 *μ*L of capsaicin (5.2 nmol/paw) or glutamate (20 *μ*mol/paw) into the right hind paw. Immediately animals were placed in individual chambers and the period in which the animal remained licking or biting the capsaicin- or glutamate-injected paw was counted during 5 or 15 minutes, respectively.

### 2.6. Evaluation of the Mechanism of Antinociceptive Action of* Pereskia bleo*

In order to investigate the possible participation of the opioid, cholinergic, and nitric oxide systems in the antinociceptive effect of E, H, EA, and B, mice were pretreated with several antagonists. Naloxone (1 mg/kg, i.p.), an opioid receptor antagonist, atropine (1 mg/kg, i.p.), a cholinergic receptor antagonist, and L-nitro-arginine methyl ester (L-NAME, 3 mg/kg, i.p.), an inhibitor of nitric oxide synthase, were administered 30 min before 100 mg/kg, p.o., of E, H, EA, and B. The choice of the doses for the antagonists or inhibitors and their treatment times were based on previous data described in the literature [14, 15] and experiments conducted in our laboratory. Dose response curves of each antagonist were previously performed and the dose that reduced by 50% the response of the agonist was chosen for these assays [9]. The nociceptive response was evaluated using the hot plate test.

### 2.7. Reduction of Spontaneous Activity

The spontaneous activity was evaluated as described in Barros et al. [16] and Figueiredo et al. [17]. Mice received oral administration of E, H, EA, or B (at 100 mg/kg, p.o.). Immediately, they were placed individually in an observation chamber in which floor was divided into 50 squares (5 cm × 5 cm). The total number of squares by which mice walked during 5 min was counted.

The effect of fractions on locomotor performance was also tested on the rotarod apparatus as described previously [17, 18]. Twenty-four hours before the experiments, all animals were trained in the rotarod (3.7 cm in diameter, 8 r.p.m.) until they could remain in the apparatus for 60 s without falling. On the day of the experiment, mice were treated with E, H, EA, or B at 100 mg/kg, p.o., and tested in the rotarod from 0.5 up to 3.5 h after their administration. The number of falls from the apparatus was recorded with a stopwatch for up to 240 s.

### 2.8. Acute Toxicity

Acute toxicity parameters were determined following the method described by Lorke [19]. Oral dose of the E, H, EA, and B (500 mg/kg) was administered to groups of ten mice (five males and five females). Parameters like convulsion, sedation, reflex, hyperactivity, increased or decreased respiration, and food and water intake were observed over a period of 5 days to analyse the behaviour of the animals. After that, mice's stomachs were removed in order to search for ulcers (single or multiple erosion, ulcer or perforation) and instances of hyperemia were counted.

### 2.9. Statistical Analysis

All results are presented as mean ± SD, and each group of animals contained 6–10 mice. The area under the curve (AUC) was calculated using Prism Software 5.0. Statistical significance between groups was determined by analyses of variance (ANOVA) followed by Bonferroni's test. Results were considered significant when *p* values were less than 0.05 (^*∗*^ *p* < 0.05).

## 3. Results

### 3.1. Effect of* Pereskia bleo* Fractions on the Hot Plate Model

A previous work from our group showed the isolation of few substances in fractions obtained from leaves of* P. bleo* as well as the anti-inflammatory and peripheral antinociceptive effects of the crude ethanol extract and its hexane, ethyl acetate, and butanol fractions [8]. Based on these results, we decided to evaluate a possible central antinociceptive effect for those same fractions and compounds and investigate a possible mechanism of action for such activity. In this regard, we used central model of pain (the hot plate) as well as the capsaicin- and glutamate-induced paw licking.

**Figure 1:**
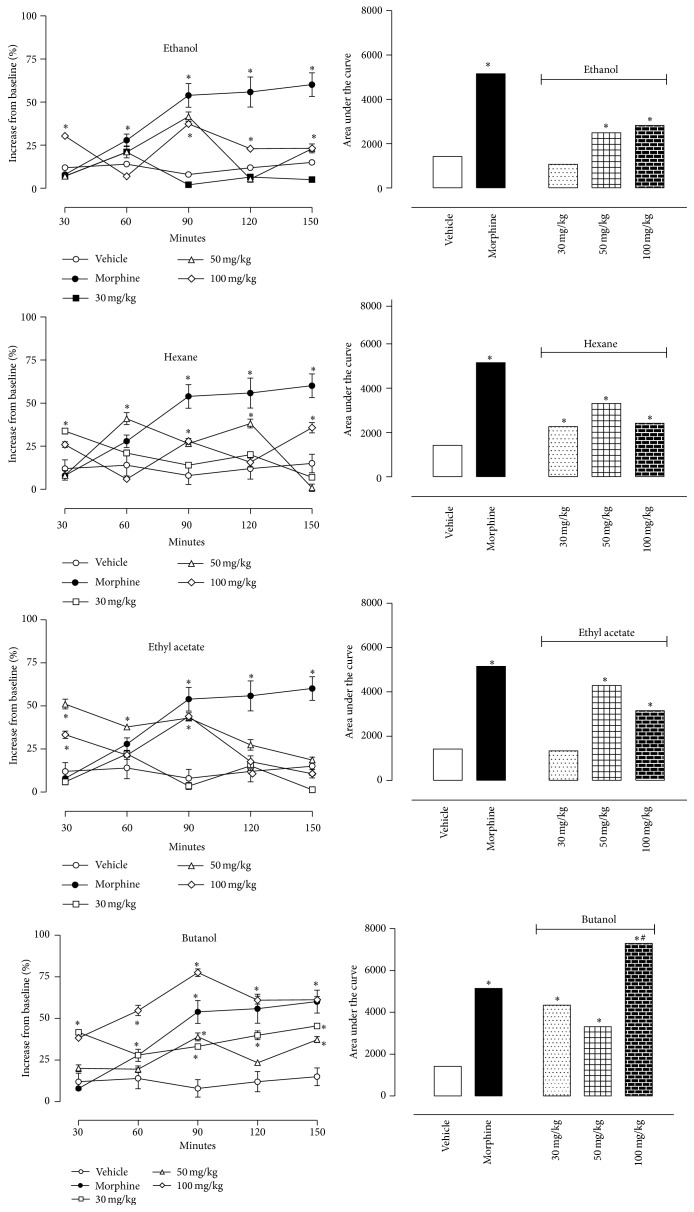
Effects of oral administration of E, H, EA, or B fractions of* Pereskia bleo* Kunth leaves on the hot plate model. Control groups were composed of vehicle or morphine (5 mg/kg, p.o.). Results are presented as mean ± S.D. (*n* = 6–10) of increase in baseline or area under the curve. Statistical significance was calculated by ANOVA with Bonferroni's test where ^*∗*^ *p* < 0.005 when comparing morphine, ethanol extract, hexane, ethyl acetate, or butanol fractions-treated mice with vehicle-treated group and ^#^ *p* < 0.005 when comparing E, H, EA, or B fractions-treated group with morphine-treated group. Where no error bars are shown, this is because the error bars are so small that they cannot be seen in the graphic.

Figure 1 shows the effects of* P. bleo* fractions on the hot plate model. The pretreatment of mice with increasing doses of E, H, EA, and B fractions led to a correspondent increase in the time that the animal took to jump after being placed in the hot plate. Pretreatment with E promoted a dose dependent increase in the antinociceptive response. This effect was maximal at 90 minutes following administration. After this, decay was observed reaching values similar to vehicle-treated group at 150 min. Similar patterns were also observed for EA and B fractions. B fraction was the most potent in developing an antinociceptive effect. All doses tested significantly increased the antinociceptive activity and this effect was comparable to that observed for morphine (at 30 and 50 mg/kg doses). At 100 mg/kg dose, the activity was significantly higher than the one observed for morphine. The antinociceptive effect appeared 30 min after the injection, reaching the peak at 90 min and persisting significantly up to 120 min. The analgesic effect of morphine was observed 60 min after administration and reached a plateau between 90 and 150 min. Converting the values of morphine results to a graph of area under the curve, a value of 5,153 is obtained for this agonist. Administration of vehicle did not show any antinociceptive response.

Isolated compounds, sitosterol and vitexin, were also tested in the hot plate model. Doses used were based on the relative amount of each substance in the fraction that they were isolated from the plant. Sitosterol represented 3.3% of the hexane fraction while vitexin represented 0.33% of the ethyl acetate fraction. Based on these values we decided on the doses to test for vitexin and sitosterol.

**Figure 2:**
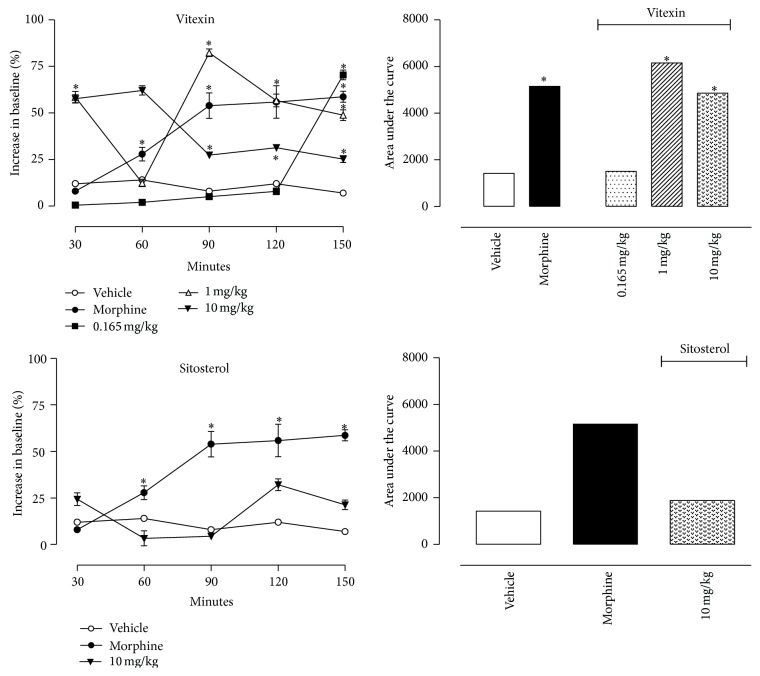
Effects of oral administration of the vitexin or sitosterol on the hot plate model. Control groups were composed of vehicle or morphine (5 mg/kg, p.o.). Results were presented as mean ± S.D. (*n* = 6–10) of increase in baseline or area under the curve. Statistical significance was calculated by ANOVA with Bonferroni's test where ^*∗*^ *p* < 0.005 when comparing morphine-, sitosterol-, or vitexin-treated mice with vehicle-treated group. Where no error bars are shown, this is because they are smaller than the symbol.

Vitexin (at 1 and 10 mg/kg) significantly increased the reaction time of mice. However, the dose corresponding to the amount found in the extract, that is, 0.165 mg/kg, did not demonstrate any effect. Sitosterol, even at 10 mg/kg dose, did not increase the mice response suggesting that this substance does not have effect in this model (Figure 2).

### 3.2. Effect of* Pereskia bleo* Fractions and Their Isolated Compounds on the Capsaicin- and Glutamate-Induced Paw Licking

**Figure 3:**
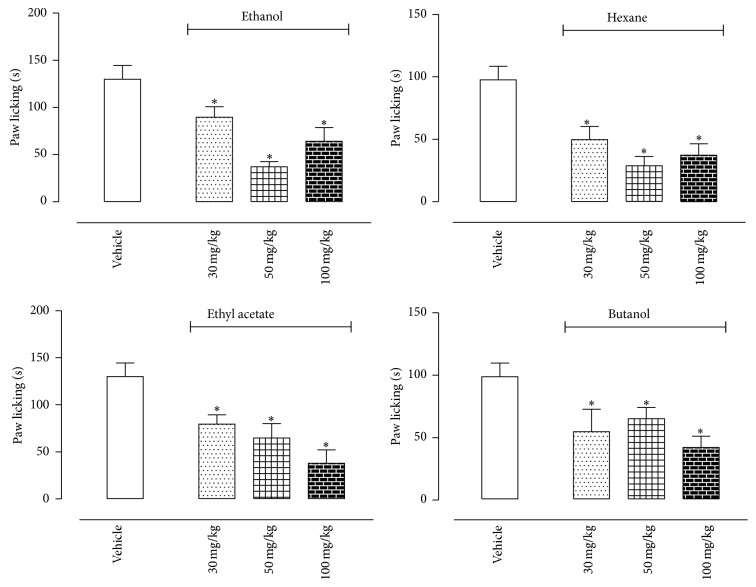
Effects of oral administration of the E, H, EA, or B fractions of* Pereskia bleo* Kunth leaves on the capsaicin-induced biting model. Control groups were composed of vehicle. Results were presented as mean ± S.D. (*n* = 6–10) of the time (in seconds) that the animal spent biting or licking the capsaicin-injected paw. Statistical significance was calculated by ANOVA with Bonferroni's test where ^*∗*^ *p* < 0.005 when comparing E, H, EA, or B fractions-treated mice with vehicle-treated group.

Injection of capsaicin (5.2 nmol/paw) in the paw resulted in a licking and biting response during 5 min after injection leading to 129.9 ± 14.7 seconds of response. Pretreatment of mice with increasing doses of fractions and their isolated compounds promoted a corresponding reduction on licking response. Maximal effects were observed with 100 mg/kg dose, which showed 50.6%, 61.7%, 70.8%, and 57.7% inhibition for E, H, EA, and B, respectively (129.9 ± 14.7 sec in vehicle-treated group versus 64.1 ± 14.7 sec, 49.8 ± 11.9 sec, 37.9 ± 14.3 sec, and 54.9 ± 11.8 sec, resp.) (Figure 3).

**Figure 4:**
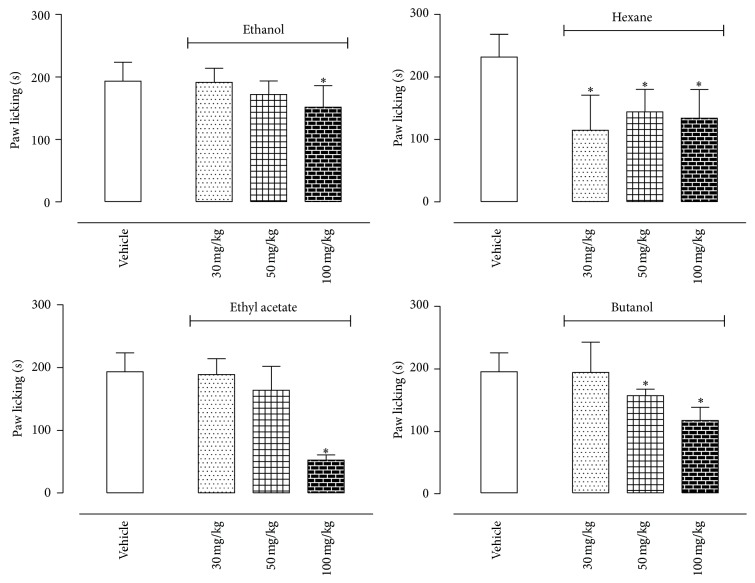
Effects of oral administration of the E, H, EA, or B fractions of* Pereskia bleo* Kunth leaves on the glutamate-induced licking model. Control groups were composed of vehicle. Results were presented as mean ± S.D. (*n* = 6–10) of the time (in seconds) that the animal spent biting or licking the glutamate-injected paw. Statistical significance was calculated by ANOVA with Bonferroni's test where ^*∗*^ *p* < 0.005 when comparing E, H, EA, or B fractions-treated mice with vehicle-treated group.

Similar to capsaicin, the glutamate (20 *μ*mol/paw) also induced a licking response after intraplantar injection that lasted until 15 min after the injection. Ethanol extract and ethyl acetate fraction reduced the glutamate-induced licking only at the highest dose (100 mg/kg). Butanol fraction developed a dose response inhibitory effect, with a significant effect at the doses of 50 and 100 mg/kg. All doses tested of the hexane fraction equally reduced the time that the animals spent licking the glutamate-injected paws (Figure 4).

**Figure 5:**
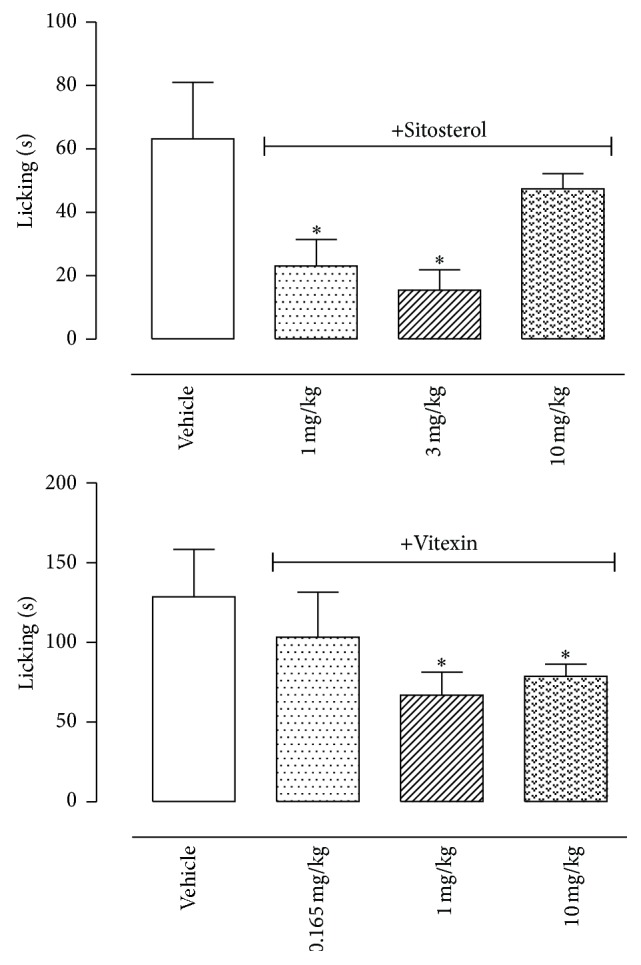
Effects of sitosterol and vitexin isolated from the H and EA fractions of* Pereskia bleo* Kunth leaves, respectively, on the capsaicin-induced biting model. Control groups were composed of vehicle. Results were presented as mean ± S.D. (*n* = 6–10) of the time (in seconds) that the animal spent biting or licking the capsaicin-injected paw. Statistical significance was calculated by ANOVA with Bonferroni's test where ^*∗*^ *p* < 0.005 when comparing vitexin- or sitosterol-treated mice with vehicle-treated group.

Results obtained in Figure 5 showed that both isolated substances significantly reduced licking response induced by capsaicin. However, only the higher doses of vitexin (1 and 10 mg/kg) significantly reduced capsaicin-licking response.

**Figure 6:**
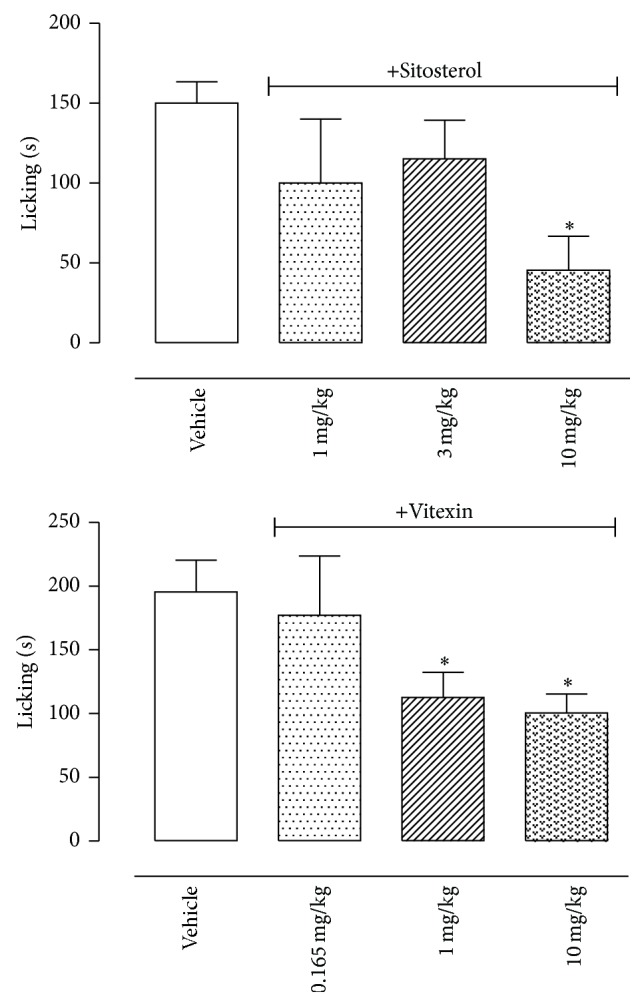
Effects of sitosterol and vitexin isolated from the H and EA fractions of* Pereskia bleo* Kunth leaves on the glutamate-induced licking model. Controls groups were composed of vehicle. Results were presented as mean ± S.D. (*n* = 6–10) of the time (in seconds) that the animal spent biting or licking the glutamate-injected paw. Statistical significance was calculated by ANOVA with Bonferroni's test where ^*∗*^ *p* < 0.005 when comparing sitosterol- or vitexin-treated mice with vehicle-treated group.

Pretreatment of mice with sitosterol or vitexin also reduced biting or licking response induced by glutamate. However, these effects were observed only with higher doses of both substances (10 mg/kg sitosterol and 1 and 10 mg/kg dose of vitexin) (Figure 6).

### 3.3. Mechanism of Action of* Pereskia bleo* Fractions

**Figure 7:**
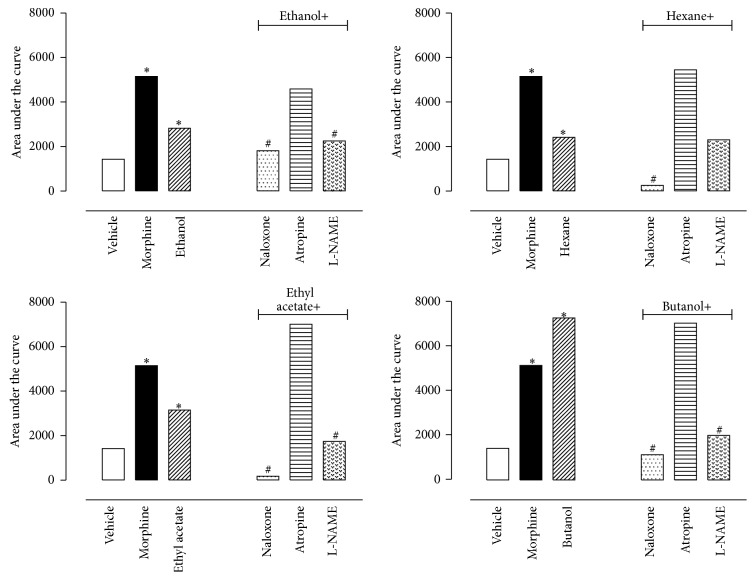
Effects of different antagonists on antinociceptive activity of E, H, EA, and B fractions of* Pereskia bleo* Kunth leaves in the hot plate model. Animals were pretreated with atropine (1 mg/kg, i.p.), L-NAME (3 mg/kg, i.p.), or naloxone (1 mg/kg, i.p.) 30 min prior to oral administration of fractions (100 mg/kg). The results are presented as mean ± S.D. (*n* = 6–10) of area under the curve calculated using Prism Software 5.0. Statistical significance was calculated by ANOVA followed by Bonferroni's test. ^*∗*^ *p* < 0.005 when comparing morphine- or fraction-treated mice with vehicle-treated group and ^#^ *p* < 0.005 when comparing antagonists treated mice with fractions-treated group.

As all fractions of* P. bleo* showed a significant effect in the hot plate model, we decided to investigate the possible mechanism by which the antinociceptive activity occurs. In this regard, animals were pretreated with an opioid receptor antagonist (naloxone, 1 mg/kg, i.p.), a cholinergic receptor antagonist (atropine, 1 mg/kg, i.p.), or an inhibitor of nitric oxide synthase (L-NAME, 3 mg/kg, i.p.), 15 min prior to oral administration of a single dose of E or fractions. The results indicate that naloxone completely prevented the antinociceptive effect of E and all fractions, mainly the H and EA ones. On the other hand, atropine did not prevent the antinociceptive effects. Conversely, it increased the effect of all fractions. L-NAME did not change the E and H effects; however, it reverted the antinociceptive effects of EA and B fractions (Figure 7).

### 3.4. Assessment of Side Effects and Toxicity

A single oral administration of E, H, EA, or B (at 100 mg/kg) did not induce significant mucosal lesion 4 h after their administration, presenting visual conditions similar to those for the vehicle (data not shown).  Table 1 shows the effects of E, H, EA, or B (100 mg/kg, p.o.) and vehicle on the rotarod test from 0.5 to 4 h. The fractions tested did not alter the fall latency or number of falls in the rotarod test when compared with the saline group. Also, none of the fractions altered the number of crossings or rearing responses when compared with the vehicle group.

## 4. Discussion

In the present study we have shown the central antinociceptive effect of* P. bleo* leaves E, H, EA, and B as well as of two isolated compounds. We were also able to identify, at least in part, the mechanism of their action. Recently, we showed that the fractions obtained from leaves of* P. bleo* presented peripheral antinociceptive effect in acetic acid-induced contortions and formalin-induced licking response [8]. We then decided to evaluate the central antinociceptive effects of this plant attempting to identify its mechanism of action. For this purpose we used the hot plate model. The paws of mice and rats are very sensitive to heat at temperatures that do not damage their skin. The animals respond with jumps or withdrawal or licking of the paws [20]. The time for these responses can be enhanced by administration of central acting analgesics. Drugs such as acetylsalicylic acid or phenylacetic acid type, with peripheral action, do not affect these responses. The hot plate test has been used by many investigators and has been found to be suitable for the evaluation of centrally but not peripherally acting analgesics [21, 22].

Our results show that all fractions obtained from* P. bleo* increased the antinociceptive response and consequently increased the baseline in the hot plate model. Although E and EA demonstrated significant result only at 50 and 100 mg/kg, H and B demonstrated significant effects at a smaller dose of 30 mg/kg. It is interesting to note that B showed results similar to morphine-treated group and that at the higher dose (100 mg/kg) its effect was significantly higher than the one observed with the opioid. It was also observed that H and EA at 100 mg/kg possessed smaller antinociceptive effect than at 50 mg/kg dose. In fact, higher doses of these fractions were less effective with a tendency to produce U-shaped responses. Such effects are described for many drugs acting at G protein coupled receptors and have been attributed to compensatory responses or conformational changes of the receptor when a certain dose of a drug is exceeded [23].

Characteristic differences occurred in the time course and maximal effects of the antinociceptive action of the fractions when comparing with morphine-treated group. A rapid onset with an early maximum effect is characteristic of the time course of action of opioid agonists (e.g., morphine), which mediate analgesia via opioid receptors under both normal and inflammatory conditions [24]. The administration of fractions produced a time course of action similar to morphine, but with less intensity. One possible explanation for the rapid onset of action might be the solubility of the substances, which allows them to rapidly reach the brain. With exception of B, which reached maximal values, all other fractions rapidly decreased the antinociceptive activity and responses returned to baseline after 150 min of administration. These results could be a consequence of an incomplete access to central areas of the brain or rapid metabolization.

To test the hypothesis that the antinociceptive effect of fractions could be due to the presence of vitexin and/or sitosterol, we also tested these isolated substances in the same model mentioned above. We observed that the vitexin treatment resulted in an antinociceptive effect in higher doses (1 and 10 mg/kg) and sitosterol did not demonstrate any effect.

Vitexin, a C-glycosylated flavone, can be found in several plants such as* Passiflora* sp.,* Crataegus* sp., bamboo leaves, pigeon pea leaves, and mung bean [25–27]. There are several papers concerning its activity as antioxidant [28, 29], antispasmodic [30, 31], anti-inflammatory [32], antiviral [33, 34], antitumor [35, 36], antimetastatic [37], antimyeloperoxidase, and anti-*Helicobacter pylori* agent [38] and inhibitor of adipogenesis [39], platelet aggregation [40], and urease [41]. The data we obtained in this work is in agreement with others of the literature demonstrating that vitexin antinociceptive effect is mediated by opioid system.

Pain sensation can be effectively controlled by systems of neurotransmitters such as oxidonitrergic, opioid, and cholinergic systems, in addition to others, acting in different ways during the pain transmission process and the interference with one of the components makes them a very interesting and complex phenomenon [42]. To study whether fractions of* P. bleo* develop their analgesic properties interfering with one of these components we evaluated the mechanism of action of the fractions pretreating the animals with some inhibitors of the systems listed above.

Among the systems involved in pain, the opioid system is one of the most important. This system participates in both the perception and modulation of the pain process by both central and peripheral mechanisms [43]. To evaluate the participation of the opioid system, mice were pretreated with naloxone, an opioid antagonist. Our data clearly demonstrated that the opioid system is intensely present in the antinociceptive activity of fractions. This observation is clearly illustrated by the fact that naloxone almost abolished the analgesic effect of all fractions. These results allowed us to suggest that the different fractions contain several substances with significant effects on opioid receptors at central nervous system.

Several studies have also demonstrated that cholinergic pathways are implicated in antinociception at the spinal level [44–47]. It is noteworthy that atropine-induced cholinergic receptor blockade is an important effect that involves medullar and supramedullar centres related to antinociception [48]. Moreover, cholinergic pain modulation may also involve first-order neurons (sensory neurons responsible for delivering sensory information to the CNS) via muscarinic acetylcholine M2 receptors. In this context, Dussor and colleagues have demonstrated that activating peripheral M2 receptors produces antinociception in vivo and inhibits antinociceptive activity in vitro [49]. In our study, we also demonstrated that systemic atropine did not influence the antinociceptive effect of all the fractions. Surprisingly, atropine increased the antinociceptive effect from E and EA. One possible explanation for these results could be that the blockage of muscarinic receptors by atropine prevented the constituents of fractions from interacting with these receptors, thus enabling them to interact with other receptors. As opioid receptors are involved in the mechanism of action of* P. bleo* fractions, it could be that the constituents of fractions that did not interact with muscarinic receptors can interact with opioid receptors. The final result could be an increase in the antinociceptive effect in the presence of atropine.

Another aspect of the antinociceptive effect of* P. bleo* investigated was its possible involvement with vanilloid receptors. It is well known that capsaicin activates specific vanilloid receptors (TRVP1) on sensory nerve endings, triggering cation influx, action potential firing, transmission of information to the CNS, and the subsequent sensation of burning pain and discomfort [50–52]. It is also well established that the TRPV1 receptor plays an important role in the development of inflammatory hyperalgesia [53–55]. Changes in levels of activation of TRPV1 expressing afferent fibres may be critical in an ascending pathway that ultimately results in the activation of descending pain modulatory systems. Such ascending and descending pathways may form part of a loop that ultimately underlies hypersensitivity or hyposensitivity in cases of hyperalgesia or after injuries. Our data indicate that TRPV1 receptors also play a role in the development of* P. bleo* fractions-induced antinociception since some fractions and the two isolated compounds reduced capsaicin-induced response.

We also examined whether glutamate receptors could be involved in the* P. bleo*-induced antinociception. Glutamate, an excitatory amino acid, is one of several neurotransmitters involved in nociception. It has an important role in sensitization of dorsal horn of spinal cord, since primary afferent fibres stimulation results in liberation of glutamate [56]. It was described that intraplantar or intrathecal injection of glutamate promotes a nociceptive response in animals with licking and biting of the injected place [13, 57]. Our results indicate that the antinociceptive effect of fractions and isolated compounds of* P. bleo* results in part of a direct modulation of NMDA or non-NMDA receptors. It also could be due to an indirect effect through other mediators of glutamatergic system. Metabotropic glutamate receptors (subtype 5) (mGlut5) are involved in thermic hyperalgesia and acute nociception [58] and in spinal nociception [59]. Sasamura and colleagues [60] also reported the presence of glutamatergic terminals sensitive to capsaicin in hypothalamus, some of them expressing TRPV1 receptors. And when these receptors were activated they evoked the glutamate release [59]. These observations could explain, at least in part, the antinociceptive effect observed with* P. bleo* fractions and isolated compounds.

We also demonstrated that vitexin and sitosterol, isolated from EA and H fractions, respectively, have significant effects reducing licking responses induced by capsaicin or glutamate. These results suggest that, at least in part, these substances may be responsible for the antinociceptive activities observed when ethyl acetate or hexane fractions were administered.

Although the doses of* P. bleo* used were higher than traditional analgesic drugs, one must take into account the fact that these fractions are not pure drugs or synthetic compounds. They have different constituents and at different concentrations. Another fact is that* P. bleo* was administered orally. The absorption by gastrointestinal tract could be influenced by the pH in the stomach and the liposolubility of the fractions constituents may interfere with the absorption.

Our results aimed to clarify the mechanism of the action of the fractions obtained from* P. bleo*,* a medicinal plant popularly used in Malaysia*.* This work also attempted the validation* of a less expensive therapeutic toll with less adverse effects to treat possible algesic processes, reinforcing the importance of* P. bleo* as a phytomedicine.

In summary, our results indicate that* P. bleo* has an antinociceptive effect that is mediated, at least in part, by opioidergic, nitrergic, and glutamatergic pathways and at least part of the effect can also be due to the presence of vitexin and sitosterol in fractions.

## Figures and Tables

**Table 1:** Effects of ethanol extract, hexane, ethyl acetate, and butanol fractions on spontaneous activity mice.

	Hour after treatment			
	0.5	1	2	4
*Spontaneous activity *				
Vehicle	55 ± 6.8	62.4 ± 7.1	59.1 ± 6.7	53.9 ± 6.4
E	60.8 ± 9.7	65.7 ± 9.9	52.4 ± 6.9	56.7 ± 8.1
H	51.9 ± 7.7	58.3 ± 8.6	55.4 ± 10.1	60.3 ± 7.2
EA	66.1 ± 11.6	61.7 ± 9.3	54.6 ± 7.8	49.8 ± 8.1
B	51.9 ± 6.9	59.9 ± 8.7	66.1 ± 7.8	48.4 ± 6.8

*Locomotor performance *				
Vehicle	12 ± 1.3	15.4 ± 3.3	14.7 ± 2.8	19.7 ± 2.6
E	8.9 ± 3.1	12.7 ± 3.1	13.9 ± 4.2	15.3 ± 4.8
H	9.9 ± 1.8	14.7 ± 2.1	16.7 ± 2.9	17.6 ± 2.7
EA	11.4 ± 3.1	16.7 ± 1.9	15.5 ± 2.4	16.8 ± 1.9
B	10.7 ± 3.1	12.5 ± 3.8	15.4 ± 4.4	21.8 ± 5.2

Results are presented as mean ± S.D. (*n* = 6–10).
